# Applications of Nanomaterials and Nanotechnology in Energy Storage Device

**DOI:** 10.3390/nano12244353

**Published:** 2022-12-07

**Authors:** Joonho Bae

**Affiliations:** Department of Physics, Gachon University, Seongnam-si 13102, Gyeonggi-do, Republic of Korea; baejh2k@gachon.ac.kr

Nanomaterials and nanotechnology have played central roles in the realization of high-efficiency and next-generation energy storage devices. The high surface-to-volume ratio of various nanomaterials allows for short diffusion pathways on the electrodes of the energy storage devices, inevitably resulting in desired merits of the devices, such as large power and energy densities. Thus, nanomaterials and nanotechnology are, unprecedentedly, shaping all energy storage device technologies and industries.

Versatile applications of nanomaterials have been demonstrated in all energy device aspects, e.g., a novel solid electrolyte was fabricated through the immobilization of an ionic liquid in the nanopores of a metal–organic framework, enhancing the performance of lithium metal batteries [1]. For applications in microbial fuel cells, carbon nanofiber/PDMS nanocomposites were successfully utilized as a corrosion-resistant coating for copper anodes [2]. Recently, the density functional theory was employed to investigate the performance of V4C3 MXene as an anode for Li-ion and Na-ion batteries [3]. A cheap electrocatalyst and possible solutions to the more serious energy generation problems were proposed through the use of compost soil as a novel electrocatalyst for ammonium fuel cells [4]. The influence of the morphology, surface area and surface modification of carbonaceous additives on the performance of corresponding cathodes was evaluated for a lithium–sulfur battery [5]. Finally, two Ni(OH)_2_ synthesis routes were discussed and their differences in mechanisms were presented for battery applications [6].

In this Special Issue of *Nanomaterials*, we present recent advancements in nanomaterials and nanotechnology for energy storage devices, including, but not limited to, batteries, Li-ion batteries, Li–S batteries, electric double-layer capacitors, hybrid capacitors and fuel cells. As the guest editor, I am very grateful for all the author contributions, and hope readers enjoy the articles found herein.
